# Internetressourcen zur Plantarfasziitis: Deutschsprachige Analyse des Informationsgehalts digitaler Quellen

**DOI:** 10.1007/s00132-025-04641-8

**Published:** 2025-03-25

**Authors:** Marianne Rosenthal, Mark Lenz, Sophie Maria Tengler, Sebastian Sachse, Matthias Walcher, Klaus E. Roth, Lena Mohr, Kajetan Klos

**Affiliations:** 1 Klinik für Unfall‑, Hand- und Wiederherstellungschirurgie und Orthopädie, Universitätsklinikum Jena, Am Klinikum 1, 07747 Jena, Deutschland; 2 OCW – Orthopädie Chirurgie Würzburg, Würzburg, Deutschland; 3 Meliva Gelenkzentrum Rhein-Main, Hochheim, Mainz, Wiesbaden, Deutschland; 4 Universitätsklinikum Mainz, Mainz, Deutschland

**Keywords:** Patientenbildung, Sporttherapie, Fußerkrankung, Fersensporn, Entzündungen, Education of patient, Exercise therapy, Foot diseases, Heel spur syndrome, Inflammation

## Abstract

**Hintergrund:**

Die Plantarfasziitis gehört zu den häufigen allgemeinmedizinischen und orthopädischen Konsultationen. Webseiten ermöglichen hier eine breite Gesundheitsbildung. Allerdings erscheint die Qualität mitunter fraglich.

**Ziel:**

Die Studie evaluiert die Qualität von Struktur, Inhalt, Verständlichkeit und Gestaltung von Webseiten zur Plantarfasziitis. Aus Ergebnissen und Fachinformation wurde ein digitaler Ratgeber zu Fußpathologien erstellt.

**Material und Methoden:**

Die Schlagwörter von Google Ads wurden in Google, Bing und Yahoo eingegeben und die Suchergebnisse mit diversen Scores (EQIP36, 25-Item, Flesch-Kincaid) bewertet. Jeder Score floss zu einem Drittel in den Gesamtscore ein. Die 3 besten Webseiten wurden in einer Umfrage getestet und eine Internetseite erstellt.

**Ergebnisse:**

137 Internetseiten und 37 Videos wurden analysiert. Die Mittelwerte betrugen 72 (EQIP36), 15 (25-Item), 43 (Flesch-Kincaid), 59 (Gesamtscore). Ärzte erstellten die meisten Webseiten mit führenden Resultaten im 25-Item. Das höchste Leseniveau erreichten Webseiten von Enzyklopädien, den größten EQIP36 und Gesamtscore die von Kliniken. Keine Quellen enthielten Webseiten mit niedrigem Gesamtscore, EQIP36 und 25-Item. Webseiten mit Videos von Fachexperten zeigten höhere Gesamtscores und Leseniveaus. Internetseiten schnitten in der Umfrage im Vergleich zur Scoreanalyse schlechter ab.

**Diskussion:**

Ein Defizit hochwertiger Webseiten zur Plantarfasziitis wurde nachgewiesen. Bei Erstellung gesundheitsbezogener Internetquellen sollten mangelhafte Aspekte verbessert werden.

## Abstract (en)

**Background:**

Plantar fasciitis belongs to the most common reasons for a consultation in medical institutions. Websites are assisting health education in this field; however, the quality is sometimes questionable.

**Objectives:**

The quality of websites thematising plantar fasciitis was evaluated in a descriptive survey regarding content, structure, readability and visual aspects. Information of literature added by score results was used to create a website describing foot diseases.

**Materials and methods:**

Keywords chosen by Google Ads were searched for in Google, Bing and Yahoo. Search results were analysed according to linguistic scores (EQIP36, 25-Item, Flesch-Kincaid). A third of these score worths were summarized as a total score. An online survey examined three websites with the highest scores. An optimized website was created.

**Results:**

137 websites and 37 videos were scored. Arithmetic averages amounted to 72 (EQIP36), 15 (25-Item), 43 (Flesch-Kincaid), 59 (total score). Major websites with the best results in 25-Item were prepared by physicians. Websites of encyclopedias reached the best reading level, whereas those of health insurance funds received the highest values in EQIP36 and the total score. Websites with the lowest total score, EQIP36, 25-Item did not mention any sources. Websites including videos created by the medical sector reached a higher total score and reading levels. The outcome of the survey was slightly worse than the results of the score.

**Conclusions:**

A lack of high qualified websites regarding foot pain was identified. This information can help producers of websites for digital patient education to improve the inadequate aspects.

**Graphic abstract:**

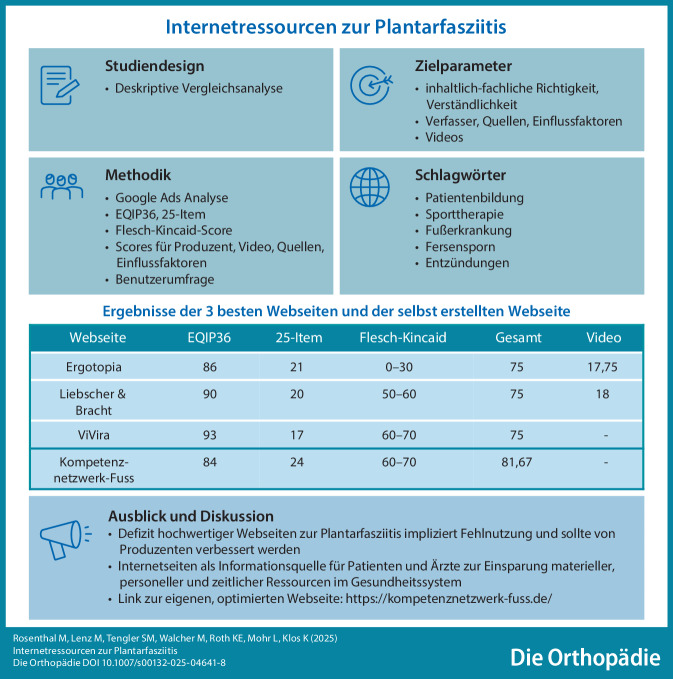

## Hinführung

Die Plantarfasziitis weist eine Prävalenz von 5–10 % [7] auf. Betroffene informieren sich aufgrund allgemeiner Verfügbarkeit und niedriger Hemmschwelle während des Krankheitsverlauf im Internet über Diagnose und Therapie. Der einfache Zugang in den Industrienationen ermöglicht eine kosten-, zeit- und ressourcenreduzierte Informationsbeschaffung, um im häuslichen Umfeld selbstständig Präventions- und Therapiemethoden durchzuführen [15, 17, 25]. Der folgende Artikel beschreibt die Qualität von Internetressourcen. Erwähnt wird eine eigene Webseite zu Fußpathologien.

## Einleitung

Die Plantarfasziitis stellt eine häufige Ursache einer orthopädischen Konsultation dar.

Ätiologisch ist sie meist mit einer chronischen Überlastung der Plantarfaszie assoziiert. Risikofaktoren umfassen Fehlstellungen wie Überpronation oder dauerhafte Überlastung durch permanentes Stehen. Auch Adipositas und Muskeldysbalancen wie Wadenkontrakturen tragen zur Pathogenese bei [2, 11].

Klinisch manifestiert sich die Erkrankung typischerweise durch intensiven Fersenschmerz, insbesondere nach Ruhephasen, wie dem morgendlichen Aufstehen. Dieser Anlaufschmerz kann sich durch weitere Belastung vorübergehend reduzieren, jedoch im Tagesverlauf erneut auftreten [3].

Die Therapie ist vielschichtig und reicht von konservativen Maßnahmen wie spezifischen Dehnübungen [12], orthopädischen Einlagen [4] und Physiotherapie bis hin zu medikamentöser Schmerztherapie [8]. In refraktären Fällen sind extrakorporale Stoßwellen [1], Injektionen und als Ultima Ratio eine chirurgische Intervention indiziert [19]. Ein essenzieller Faktor ist die Patientenedukation und Anleitung zu Eigenübungen. Ein geeignetes Medium hierfür stellt das Internet dar, auch zur Eigenrecherche vor dem Arztbesuch. Hochwertige Informationsressourcen hätten somit das Potenzial, die Behandlung der Plantarfasziitis zu optimieren und Arztkontakte zu reduzieren.

Diese Arbeit hat zum Ziel, die Qualität von Internetseiten zur Plantarfasziitis zu erfassen und basierend auf den Ergebnissen eine optimierte Webseite zu erstellen.

## Methodik

### Schlagwortsuche, Webseitenwahl

**Abb. 1:**
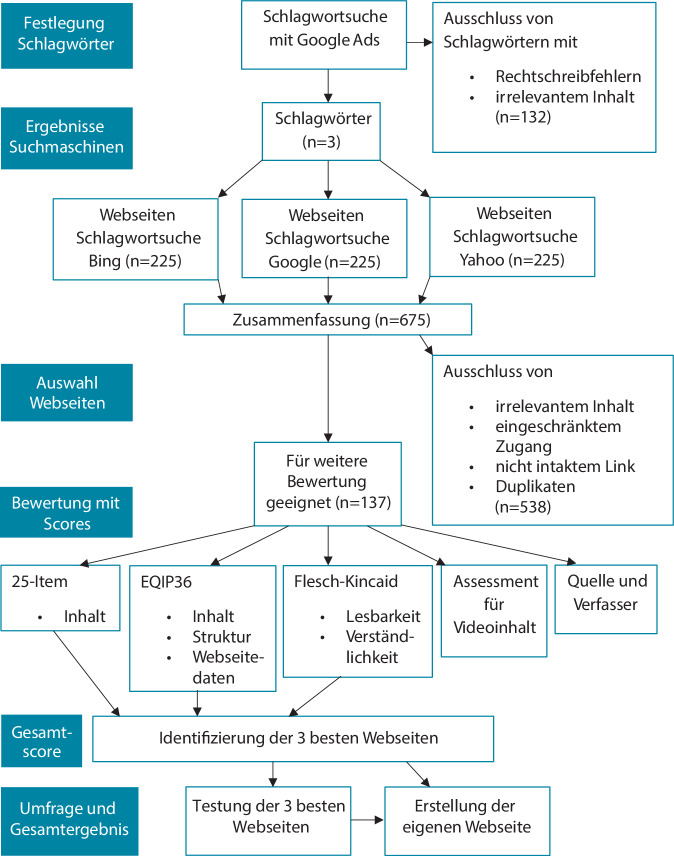
Fließschema zum Vorgehen

Abb. 1 gibt einen Überblick über das methodische Vorgehen. Die Suchbegriffe „Fersenschmerz“, „Fersensporn“ und „Plantarfasziitis“ wurden mit Google Ads im Februar 2023 ausgewählt. Mit dieser Werbeplattform lassen sich häufig verwendete Schlagwörter sowie zusätzliche Daten identifizieren. Anschließend wurden die Schlagwörter in die 3 am häufigsten verwendeten Suchmaschinen Google (80 %), Bing (12 %) und Yahoo (3 %) eingegeben [18] und die 25 ersten Suchergebnisse ausgewählt.

Internetseiten mit abweichendem Inhalt, beschädigten Links, eingeschränktem Zugang, reinem Videoinhalt und primär kommerzieller Prägung wurden von der Analyse ausgeschlossen.

### Inhalt

Diese Analyse erfolgte anhand des auf Fachinformation basierenden, modifizierten 25-Item-Scores [20]. Der 25-Item-Score wurde an das Krankheitsbild angepasst und umfasst 25 Kriterien zu Ätiologie, Symptomatik, Diagnose und Therapie (Bewertung von 0–100 Punkten). Für die Gesamtwertung wurde der Scorewert mit 4 multipliziert (Gesamtwert bis 100 Punkte). Die zweite Inhaltsprüfung mit dem Score „Ensuring quality infomation for patients 36“ (EQIP36) betrachtet neben dem Inhalt die Struktur, Sprache, Grafiken, Verständlichkeit und Identifikationsdaten der Webseite. Das Ergebnis des EQIP36 wurde mit der Formel $\text{EQIP}-\text{Score}=\frac{\text{`ja`}\:\mathrm{x}100}{36-\text{`irrelevant`}}$berechnet [6] und liefert Werte zwischen 0 und 100.

### Lesbarkeit, Verständlichkeit

Der Flesch-Kincaid (FK-Score) ermittelt das Bildungsniveau, das ein Leser benötigt, um den Inhalt zu erfassen. Während hohe Werte (Scorewerte > 60) eine einfache Verständlichkeit bedeuten, sind hoher Bildungsgrad und schwere Lesbarkeit mit niedrigen FK-Werten assoziiert. Zur Webseitenanalyse wurde ein Internettool des FK-Scores verwendet [14]. Da der Test eine Spannweite zwischen zwei Zahlen liefert (Gesamtwert bis 100 Punkte), wurde der Mittelwert von Minimum und Maximum für Analyse und statistische Auswertung verwendet.

### Zusammenfassung zum Gesamtscore

Die Ergebnisse der o. g. Scores (bzw. der vervierfachte 25-Item) wurden je als Drittel zu einem Gesamtscore summiert.

### Quelle, Verfasser

Die Quellen wurden in die Kategorien Fachliteratur, Artikel und Reviews, weitere Webseiten, Studien, Interviews und fehlende Quelle eingeteilt. Die Verfasser wurden in die Gruppen in Abb. 5 eingeordnet.

Zusätzlich wurden Verlinkung der Webseite und Einflussfaktoren auf den Leser erfasst. Zu den Einflussfaktoren zählten Sponsoren, webseiteneigene, kommerzielle Angebote und Links zu Experten. Bei der Verlinkung wurden die Nennung sozialer Netzwerke, Links zu Diagnose und Therapie sowie Verweise auf webseiteneigene, nicht kommerzielle, externe (nicht) kommerzielle Informationsmöglichkeiten beurteilt.

### Videos

Die 37 Videos von 16 Webseiten wurden anhand des „Assessment zur Videobewertung“ evaluiert. Der Score wurde mithilfe bestehender Videobewertungsinstrumente (Educational Suitability Assessment, JAMA- und DISCERN-Kriterien) sowie diverser Reviews [5, 17, 26] erstellt. 3 Videos wurden wegen abweichenden Inhalts bzw. Duplikation ausgeschlossen. Die Hypothese, ob Webseiten mit Videos verständlicher und umfassender sind, wurde durch Vergleich der Durchschnittswerte von EQIP36, 25-Item sowie FK von Webseiten mit und ohne Videos überprüft. Es wurden Anzahl der Ansichten und Gefällt-mir-Angaben analysiert.

### Benutzerumfrage

Aus den Scoreergebnissen wurde eine Umfrage zur Bewertung der 3 besten Internetseiten inklusive Videos durchgeführt, die sich am 25-Item orientierte. Anschließend erfolgte der Vergleich von Umfrage- und Scoreergebnissen. Score-Items, die sich auf ähnliche Inhalte bezogen, wurden zusammengefasst und der Mittelwert als Vergleichskriterium herangezogen. Demografische Daten wurden erhoben. Die Umfrage wurde mit der Onlineplattform SoSci Survey erstellt und veröffentlicht.

### Erstellung der Internetseite

Die Webseite basiert auf Fachartikeln und Analyseergebnissen. Die Inhalte wurden an 25-Item und EQIP36 angepasst. Für das Leseniveau wurde ein FK-Level und eine Lesbarkeit von > 60, ein Sprachniveau B1 (angemessen für berufliche und alltägliche Themen) und eine Lesedauer < 8 min festgelegt. Um eine für Patienten verständliche Sprache zu erzielen, wurde der Text mehrfach mit dem Textanalysetool Wortliga modifiziert. Videos und Bilder wurden im April 2024 von den Autoren aufgenommen und nach den Kriterien des o. g. Scores angepasst.

## Ergebnisse

Es wurden 137 von 225 Webseiten und 37 Videos als zusätzlicher Bestandteil der Webseiten eingeschlossenen.

### Inhalt

**Abb. 2:**
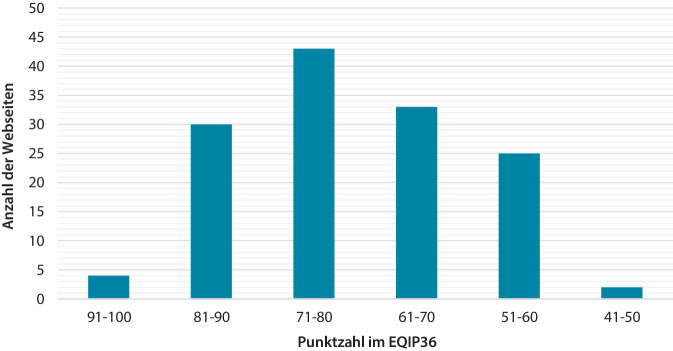
EQIP36 (Ensuring quality infomation for patients 36): Bewertung von Inhalt, Struktur, Sprache, Grafik, Verständlichkeit, Identifikationsdaten der Webseite. Je höher der Wert, desto bessere Bewertung

Der Mittelwert des EQIP36 betrug 71,78 ± 5 (Abb. 2). 39,42 % der Webseiten wiesen irrelevante, uneindeutige Grafiken auf, während nur 23,36 % wichtige, exakte Abbildungen zeigten. Struktur und Gliederung waren nachvollziehbar und anschaulich (83,94 %). Bei einem Drittel der Webseiten wurde kein Erstellungs- oder Änderungsdatum registriert. 78,83 % der Webseiten verwendeten Fachwörter für Arzneimittel und Medizinprodukte. Der Informationsgehalt wurde als vollständig eingestuft. Quantitative Vorteile (68,61 %) und Nachteile (75,91 %) wurden meist nicht nachgewiesen.

**Abb. 3:**
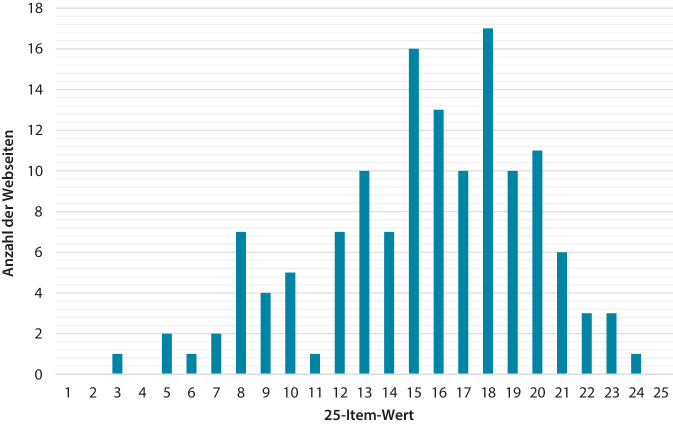
25-Item: Bewertung des Inhalts

Der Mittelwert des 25-Item betrug 15,45 ± 4,31 (Abb. 3). Die meisten Webseiten beinhalteten Dehn- (86,86 %) und Kräftigungsübungen (69,34 %). 81,75 % der Webseiten wiesen auf Vorteile von Schuhen, Einlagen oder Orthesen hin. Anatomie (83,94 %) sowie Schmerzlokalisation (89,78 %) und Risikofaktoren (89,05 %) wurden beschrieben. 47,45 % der Internetseiten beinhalteten keine Angaben zur Diagnostik.

### Verständlichkeit, Lesbarkeit

**Abb. 4:**
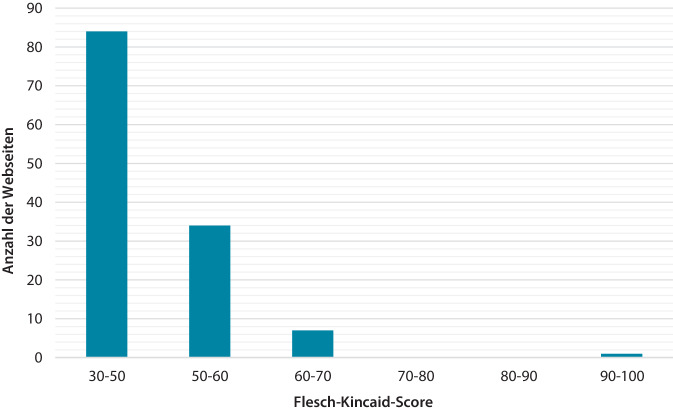
Flesch-Kincaid: Bewertung von Verständlichkeit bzw. für Verständnis nötiges Bildungsniveau. Je höher der Flesch-Kincaid, desto verständlicher bzw. desto niedriger das Leseniveau

Die Webseiten waren in ihrer Verständlichkeit heterogen (Abb. 4). Mit einem Mittelwert von 43,39 ± 12,19 entspricht die durchschnittliche Verständlichkeit von 30–50 dem Bildungsgrad eines Hauptschulabschlusses und einem schwierigen Leseniveau.

### Die 3 besten Webseiten

ViVira, Liebscher & Bracht und Ergotopia wiesen die höchsten Gesamt- und Einzelscores auf und wurden für die Umfrage verwendet.

### Verfasser, Quelle

**Abb. 5:**
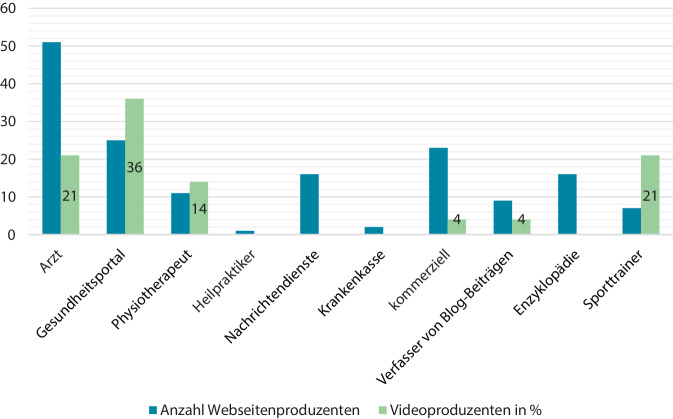
Anzahl der Webseiten nach Beruf des Erstellers und Anteil der Videoproduzenten

Ärzte erstellten 51 Webseiten, während Abb. 5. weitere Verfasser zeigt.

Bei 74 Internetseiten fehlten Quellen. 29,2 % nutzten Publikationen oder Reviews, 18,98 % Fachliteratur als Quelle.

Alle Webseiten lieferten Hinweise auf Einflussfaktoren. 50 Webseiten verwiesen auf Fachpersonen (Ärzte, Physiotherapeuten). Webseiteneigene, kommerzielle Angebote fanden sich bei 46 Internetseiten, wohingegen lediglich 3 Webseiten Sponsoren angaben. Auch soziale Netzwerke (82) und weiterführende webseiteneigene, nicht kommerzielle Informationsquellen (125) wurden verlinkt. Links zu Erläuterungen bezüglich Diagnose und Therapie sowie Verweise auf externe Informationsquellen konnten ebenfalls nachgewiesen werden.

### Videos

Der medizinische Sektor erstellte 71 % der 37 Videos. Weitere Videoproduzenten sind in Abb. 5 festgehalten. Von Sporttrainern entworfene Sequenzen wiesen umfangreiche Dehn- und Kräftigungsübungen auf. In 14 Videos fehlte der Moderator für Erläuterungen.

Die Videos von Physiotherapeuten (34,82 %) und Ärzten (29,66 %) wurden am häufigsten aufgerufen. Die Ansichtszahl korrelierte mit der Anzahl der Likes, wobei in absteigender Reihenfolge Physiotherapeuten (34,83 %) die meisten Gefällt-mir-Angaben erhielten, gefolgt von Ärzten (29,66 %). Die Scoreergebnisse von Internetseiten mit und ohne Videos unterschieden sich geringfügig. Webseiten mit Videos wiesen höhere Gesamtscores und EQIP36 auf, wohingegen Webseiten ohne Videos höhere 25-Item erreichten. Der FK von Internetseiten mit und ohne Videos war vergleichbar.

### Vergleich von Scorewerten mit Verfasser

Webseiten kommerzieller Betreiber und Nachrichtendienste erreichten den geringsten EQIP36. Internetseiten von Bloggern und Sporttrainern zeigten den niedrigsten FK (Bildungslevel: Student bzw. 10.–12. Klasse). Webseiten von Ärzten wurden im 25-Item (18,88) und Flesch-Kincaid (40,1) am besten bzw. zweitbesten bewertet. Das anspruchsvollste FK-Level wurde bei Webseiten von Enzyklopädien (38,13) nachgewiesen. Webseiten von Krankenkassen wurden im EQIP36 (85,5) und Gesamtscore (65,17) am besten evaluiert. Die Kategorien Physiotherapie, Heilpraxis und Pharmazeuten zeigten in allen Scores Durchschnittswerte.

### Abhängigkeit Gesamtscore von Quellen, Einflussfaktoren, Verlinkung

Die höchsten Gesamtscores erreichten Webseiten mit Fachliteratur als Quelle (61,6), gefolgt von jenen mit Bezug zu weiteren Webseiten und Publikationen. Webseiten mit Basis auf Fachliteratur oder Publikationen erzielten den besten EQIP36 (77,38; 77,75) und einen hohen 25-Item (17,38; 15,35). Der FK wurde bei Internetseiten mit Fachgesellschaften als Quelle am einfachsten (44) und bei Webseiten mit Bezug zu Fachbüchern am niveauvollsten (37,88) bewertet. Internetseiten ohne Quellen wurden im Gesamtscore (56,8), EQIP36 (68) und 25-Item (14,75) am niedrigsten evaluiert.

Den höchsten Gesamtscore erreichten Webseiten mit weiterführenden Links, während die mit Hinweis auf Sponsoren oder Ärzte und Physiotherapeuten den höchsten EQIP36 zeigten. Das höchste Leseniveau (FK = 30–50 bzw. Bildungslevel Student) bestand bei Internetseiten mit Hinweis auf Sponsoren. Den geringsten 25-Item erhielten Webseiten mit weiterführenden Links zu webseitenexternen Diagnose- und Therapiemöglichkeiten. Der niedrigste EQIP36 fand sich bei Webseiten mit Verlinkung sozialer Netzwerke.

### Auswertung der Benutzerumfrage

**Abb. 6:**
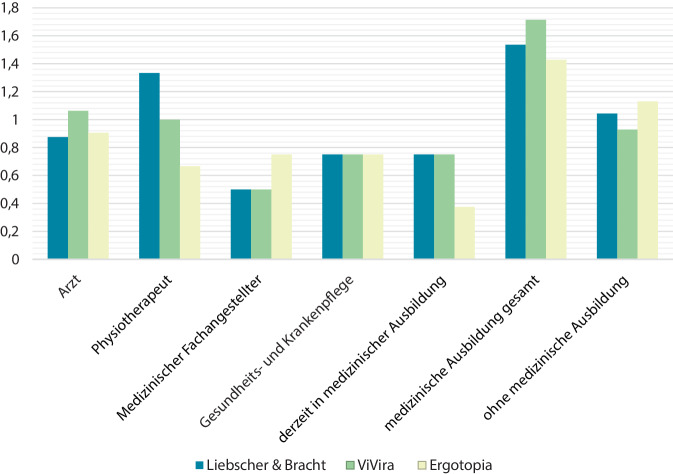
Umfrage: Bewertung der 3 besten Webseiten nach medizinischer Ausbildung

Knapp 50 von 102 Teilnehmern (42 Männer, 35 Frauen, 25 keine Angabe) beantworteten die meisten Fragen, wobei viele fehlende Antworten die Videobeurteilung betrafen. Für ein repräsentatives Gesamtbild wurden alle Teilnahmen in die Auswertung eingeschlossen. Die beste Gesamtwertung erreichte ViVira (1,9/5), gefolgt von Ergotopia (1,71) und Liebscher & Bracht (1,52/5). Abb. 6 zeigt die Bewertung durch Umfrageteilnehmer verschiedener medizinischer Ausbildungsgrade. Insgesamt wurden die Webseiten, insbesondere ViVira, von zuletzt genannten besser bewertet. Die beliebteste Webseite bei den Personen ohne medizinische Ausbildung stellte Ergotopia dar. Die meisten Befragten nutzten Arztbesuche (54) und Suchmaschinen als Informationsquelle (50), gefolgt von Fachliteratur (46), Bekanntenkreis (25), sozialen Netzwerken (4), Artificial Intelligence (2) und weiteren Informationsressourcen (5). Nutzer digitaler Suchmaschinen bewerteten die Webseiten besser als Umfrageteilnehmer, die keine digitalen Internetressourcen nutzten. Die Scorebewertung lieferte eine höhere Gesamtwertung und Bewertung der Einzelpunkte. Die beste Übereinstimmung zwischen Score und Umfrage wies ViVira auf. Auch Ergotopia zeigte eine befriedigende Übereinstimmung mit der Gesamtwertung. Hervorzuheben ist der Vergleich von Videos (69,41 %) und Bildern (75,12 %) dieser Informationsquelle. Mit 40,58 % fiel der Vergleich von Score und Umfrage bei Liebscher & Bracht aus. Der beste Konsens bezog sich auf Erklärungen zu Diagnostik und Therapie (43,87 %), wohingegen der Aufbau die geringste Übereinstimmung erbrachte (30,64 %).

### Analyse der eigenen Webseite

Zur objektiven Kontrolle der Verständlichkeit der Webseite erfolgte deren Analyse mit dem Textanalysetool Wortliga. Lesbarkeit (76), Sprachniveau (B1) und -melodie (kurz) entsprachen den festgelegten Kriterien. Der FK-Index wurde mit 61 erreicht. Satzlänge (10,8 Wörter je Satz) und Lesezeit (6:02 min) entsprachen den Vorgaben.

## Diskussion

Wie in anderen Publikationen [9, 20, 21, 23] wurden eine starke Heterogenität und ein Defizit an Internetressourcen mit fachlich korrektem Inhalt, verständlichen Formulierungen, Struktur sowie Darstellungen nachgewiesen. Viele Webseiten dienen kommerziellen und werbetechnischen Zwecken und enthalten Mängel in Bezug auf hochwertige Informationen zu Therapien, Komplikationen und Prävention. Diese Schwachstellen können sich negativ auf Verlässlichkeit und Qualität der Internetseite auswirken [22]. Die eingeschränkte Validität dieser Art der Patientenedukation kann somit durch Fehlinformation negative Konsequenzen zur Folge haben. Die Umfrage zeigte, dass digitale Internetressourcen eine beliebte, häufig genutzte Quelle für Betroffene bilden, jedoch in ihrer Qualität verbesserungswürdig sind. Dies stellt Webseitenproduzenten vor zukünftige Herausforderungen.

Folgende Faktoren limitieren die Aussagekraft der Studie. Die Auswertung stellt aufgrund der Aktualisierung der Webseiten eine Momentaufnahme dar. Durch die begrenzte Seitenzahl könnten gut aufgearbeitete Webseiten der Folgeseiten der Internetsuche unberücksichtigt geblieben sein. Diese Beschränkung wurde aufgrund der Tatsache akzeptiert, dass 70 % der Ratsuchenden lediglich die Webseiten der ersten Seiten der Internetsuche lesen [16]. Trotz der Schlagwortauswahl mithilfe von Google Ads könnten Internetnutzer auch andere Schlagwörter gesucht haben.

Die Limitation der Scores, insbesondere des EQIP36, besteht in der Allgemeingültigkeit für Patienteninformationen und reduzierter Spezifität für die Thematik. Der Score umfasst jedoch wichtige Punkte, die eine Webseite für eine reliable Gesundheitsaufklärung beinhalten soll und wurde in medizinischen Publikationen bereits angewendet [9, 10, 20]. Der 25-Item wurde aufgrund dessen an die Fragestellung angepasst. Da alle Webseiten lediglich von einer Person ausgewertet wurden, war die Verwendung standardisierter, validierter Scores notwendig, um subjektive Beurteilungsfehler zu minimieren.

Im Rahmen der Umfrage kann bei Teilnehmern der Ankereffekt [24] auftreten. Sollte die erste Webseite bereits den Präferenzen des Umfrageteilnehmers entsprechen, können Informationen oder positive Aspekte der anderen Internetseiten reduziert bis gar nicht registriert worden sein. Durch den Halo-Effekt [13] können Webseiten mit ärztlichen Autoren fälschlicherweise als besser bewertet worden sein. Eine schlechtere Bewertung durch abnehmende Konzentration oder Motivation kann v. a. die zuletzt beurteilten Webseiten betreffen. Aufgrund des Durchschnittsalters und des hohen Anteils von Medizinern bilden die Umfrageteilnehmer kein repräsentatives Gesamtbild der Bevölkerung ab. Da viele Teilnehmer bereits unter Fersenschmerzen litten, wird dennoch davon ausgegangen, dass die gewünschte Zielgruppe erreicht wurde. Die fehlenden Antworten bei der Umfrage erklären die starke Diskrepanz zwischen Umfrage- und Scorebewertung.

## Ausblick

Die Hauptaspekte für eine erfolgreiche Patientenbildung (Korrektheit, Informationsgehalt, Verständlichkeit) weisen eine heterogene Qualität zwischen den Webseiten auf [22]. Die festgestellten Mängel wurden bei der Erstellung der Webseite beachtet und verbessert. Ein Schwerpunkt wurde auf Videos gelegt, um Verständlichkeit und Anschaulichkeit zu optimieren. Diese Internetseite ist unter Kompetenznetzwerk-Fuss.de aufrufbar.

## Fazit für die Praxis

- Die Plantarfasziitis stellt eine häufige Erkrankung dar.
- 90 % der Patienten genesen durch konservative Therapien.
- Internetseiten sind schnell und einfach verfügbar und bieten sich als Informationsquelle für Patienten an.
- Das Defizit valider Webseiten erfordert deren Verbesserung hinsichtlich Informationsgehalt, Struktur und Verständlichkeit.
- Ärzte sollten auf evidenzbasierte Webseiten verweisen, um Patienten umfassend aufzuklären sowie Zeit und Kosten im Gesundheitssystem zu senken.

## Abkürzungen

EQIP36: Ensuring Quality Information for Patients
FK: Flesch-Kincaid
